# Evaluation of risk factors for 14-day and 30-day mortality among treatment regimens against *Pseudomonas aeruginosa* resistant to carbapenem but susceptible to traditional antipseudomonal non-carbapenem β-lactam agents

**DOI:** 10.1371/journal.pone.0313944

**Published:** 2024-11-19

**Authors:** Suvadee Supreeyasakon, Jantima Traipattanakul, Jatapat Hemapanpairoa, Piraporn Juntanawiwat, Wichai Santimaleeworagun

**Affiliations:** 1 College of Pharmacotherapy Thailand, Nonthaburi, Thailand; 2 Department of Pharmaceutical Care, Faculty of Pharmacy, Huachiew Chalermprakiet University, Samut Prakan, Thailand; 3 Division of Infectious Diseases, Department of Internal Medicine, Phramongkutklao Hospital and College of Medicine, Bangkok, Thailand; 4 Department of Pharmaceutical Care, Faculty of Pharmacy, Silpakorn University, Nakorn Pathom, Thailand; 5 Division of Microbiology, Phramongkutklao Hospital, Bangkok, Thailand; 6 Pharmaceutical Initiative for Resistant Bacteria and Infectious Disease Working Group (PIRBIG), Nakorn Pathom, Thailand; Nitte University, INDIA

## Abstract

*Pseudomonas aeruginosa* associated with hospital-acquired infection is often resistant to various antibiotics and is associated with high mortality worldwide. The appropriate treatment of *Pseudomonas aeruginosa* resistant to carbapenems but susceptible to traditional antipseudomonal non-carbapenem β-lactam agents (Car-R/NonCar-S *P*. *aeruginosa*) remains unclear. This retrospective study evaluated risk factors for 14-day and 30-day mortality among treatment regimens against Car-R/NonCar-S *P*. *aeruginosa*. This study enrolled 180 patients with Car-R/NonCar-S *P*. *aeruginosa* infection at Phramongkutklao Hospital between January 2019 and December 2023. The 14-day and 30-day mortality rates were 18.3% and 28.9%, respectively. Bloodstream infection (OR 1.97, 95% CI 0.88–4.43), septic shock (OR 3.3, 95% CI 1.30–8.40), Acute Physiology and Chronic Health Evaluation (APACHE) II < 14 (OR 0.13, 95% CI 0.03–0.54), Sequential Organ Failure Assessment (SOFA) <7 (OR 0.25, 95% CI 0.11–0.56), and Pitt bacteremia score <4 (OR 0.16, 95% CI 0.05–0.47) were associated with 14-day mortality. There was a higher 14-day and 30-day mortality in patients treated with piperacillin/tazobactam or aminoglycosides but there was no significant difference among antipseudomonal antimicrobial agents in the treatment of Car-R/NonCar-S *P*. *aeruginosa* infection. We supported the use of traditional antipseudomonal β-lactam agents to treat Car-R/NonCar-S *P*. *aeruginosa* infections, however the use of piperacillin/tazobactam might be concerned in some cases and further investigations were needed.

## Introduction

Infection with *Pseudomonas aeruginosa* often occurs in healthcare settings, particularly in the immunocompromised or in patients treated with an invasive medical device [1]. Multidrug resistance is common in *P*. *aeruginosa*, resulting in high mortality worldwide. Carbapenem-resistant *P*. *aeruginosa* is included in the high group on the World Health Organization (WHO)’s list of priority pathogens posing the greatest threat to human health [2]. Additionally, carbapenems were also on the WHO AWaRe Classification, which means it should be monitoring as a target of stewardship program [3].

In 2017, the 14-day and 30-day mortality rates of carbapenem-resistant *P*. *aeruginosa* infections were reported to be 19% and 30%, respectively [4]. It was also shown that the 30-day mortality rate in patients infected with *P*. *aeruginosa* differed markedly between those with healthcare-associated carbapenem-sensitive *P*. *aeruginosa* (22.6%) and its carbapenem-resistant counterpart (60.1%) [5], highlighting the threat posed by carbapenem resistance in this species. Given this high mortality rate, various studies [4, 6–12] have evaluated factors associated with mortality resulting from *P*. *aeruginosa* infection, including admission to an intensive care unit (ICU), comorbidity, treatment with an invasive medical device, site of infection, severity of infection, and the appropriateness of the treatment.

Infections due to *P*. *aeruginosa* are often problematic given the frequency of inherent or acquired resistance to various antimicrobial agents [1]. According to data from the National Antimicrobial Resistance Surveillance Center (NARST) in Thailand [13], between January and December 2022, *P*. *aeruginosa* was found to be resistant to the antipseudomonal β-lactam agents imipenem, ceftazidime, and piperacillin/tazobactam at rates of 20.8%, 19.1%, and 13.6%, respectively, with these rates being higher than those in previous years. The mechanism by which *P*. *aeruginosa* achieves carbapenem resistance differs from that in Enterobacteriaceae. Specifically, *P*. *aeruginosa* predominantly becomes resistant via decreased expression of porins and overexpression of efflux pumps, along with the production of low levels of metallo‐β‐lactamases and AmpC β‐lactamase [14, 15]. Against this background, traditional antipseudomonal β-lactams could be prescribed for treating such infections [15–17].

According to current Infectious Disease Society of America (IDSA) guidance [18], traditional β-lactam agents are the treatment of choice for carbapenem-resistant *P*. *aeruginosa* (CRPA) infections. However, at present, there is no strong evidence to confirm the superiority of any particular treatment choice. A few retrospective studies [1, 6, 12, 19] also found no difference among available antimicrobials in the treatment of *Pseudomonas aeruginosa* resistant to carbapenems but susceptible to traditional antipseudomonal non-carbapenem β-lactam agents (Car-R/nonCar-S *P*. *aeruginosa)*.

Given that there is such limited evidence about the optimal Car-R/nonCar-S *P*. *aeruginosa* treatment, the present study investigated risk factors for 14-day and 30-day mortality among treatment regimens against Car-R/NonCar-S *P*. *aeruginosa*.

## Materials and methods

### Study design and population

This retrospective study was conducted from 10 August 2023 to 31 December 2023 at a 1200-bed Phramongkutklao hospital in Bangkok, Thailand. This study was approved by the Institutional Review Board of the Royal Thai Army Medical Department at Phramongkutklao College of Medicine and Phramongkutklao Hospital (approval number: Q011h/66). Data collected after ethics approval and permission from the Director of Phramongkutklao Hospital. The informed consent was waived by the Institutional Review Board, due to retrospective design and deidentification of patient data. The patients’ characteristics, microbiological data, and clinical treatment outcomes were derived from medical records, for the period from January 2019 to December 2023. Patients were enrolled in this study if they met the following inclusion criteria: 1) aged 20 years or older, 2) infected with Car-R/Noncar-S *P*. *aeruginosa* in accordance with the Centers for Disease Control and Prevention/National Healthcare Safety Network (CDC/NHSN) surveillance definitions for specific types of infections [20], 3) receiving antipseudomonal antibiotics to which Car-R/Noncar-S *P*. *aeruginosa* is susceptible for at least 48 h, and 4) having complete medical records.

### Source data and outcomes

The following patients’ data were obtained from electronic medical records: 1) demographic data such as age, hospitalization unit, Charlson Comorbidity Index (CCI), and treatment with an invasive medical device; 2) infection data such as site of infection, antimicrobial susceptibility, coinfecting organisms, antimicrobial treatment (drug, dosing regimen), and duration until appropriate antibiotic treatment; 3) severity of illness based on septic shock, Acute Physiology and Chronic Health Evaluation (APACHE) II score, Sequential Organ Failure Assessment (SOFA) score, and Pitt bacteremia score (PBS) at the onset of infection; and 4) treatment outcomes, including 14-day and 30-day mortality. The primary outcome was factors associated with 14-day mortality.

### Definitions

The pathogen defined as *Pseudomonas aeruginosa* resistant to carbapenems but susceptible to traditional antipseudomonal non-carbapenem β-lactam agents (Car-R/NonCar-S *P*. *aeruginosa*) was resistant to imipenem or meropenem, but susceptible to at least one traditional antipseudomonal noncarbapenem β-lactam agent, including ceftazidime, cefepime, cefoperazone/sulbactam, or piperacillin/tazobactam, as interpreted by the Clinical and Laboratory Standards Institute (CLSI) guideline [21]. Isolates classified as “susceptible” or “intermediate” were considered susceptible.

Septic shock was defined when a patient received vasopressor and had a serum lactate level of ≥ 2 mmol/L. Polymicrobial infection was defined as the presence of other organisms in the same specimen from which Car-R/NonCar-S *P*. *aeruginosa* was isolated. Treatment with an invasive medical device was defined for patients treated with at least one such device, such as a nasogastric tube, Foley’s catheter, or a central venous catheter, on the day of infection.

Traditional antipseudomonal β-lactams were defined as traditional β-lactams used to treat *P*. *aeruginosa* infection, including ceftazidime, cefepime, cefoperazone/sulbactam, and piperacillin/tazobactam. Traditional antipseudomonal cephalosporins were defined as traditional cephalosporins used to treat *P*. *aeruginosa* infection, including ceftazidime, cefepime, and cefoperazone/sulbactam. Novel antipseudomonal cephalosporins were defined as newer cephalosporins used to treat *P*. *aeruginosa* infection, including ceftazidime/avibactam and ceftolozane/tazobactam.

Appropriate treatment was defined as the administration of at least one active antipseudomonal antibiotic, according to susceptibility testing, and the administration of an appropriate dose within 48 h after susceptibility was reported. The 14-day and 30-day mortality was counted from first day of diagnosis of Car-R/NonCar-S *P*. *aeruginosa* infection until death.

### Statistical analysis

The statistical software SPSS for Windows Version 27 (IBM Corp., Armonk, NY, USA) was used for analysis. Patient data and clinical characteristics were analyzed using descriptive statistics. Continuous data were compared using Student’s t-test or Mann–Whitney U test. Pearson’s chi-squared test or Fisher’s exact test was used to compare categorical variables. Logistic regression analysis was used to determine factors associated with 14-day and 30-day mortality. Variables with a *p* value of ≤0.1 in the univariate analysis were included for backward stepwise multivariate analysis. A *p* value of ≤0.05 was considered statistically significant.

## Results

### Demographic and infection characteristics

A total of 180 patients with a positive culture from January 2019 to December 2023 who met the inclusion criteria were eligible for this study. The median age was 73 years (IQR, 60–83 years), 65.6% were male, 13.3% had septic shock, 83.3% had been treated with an invasive medical device, and 29.4% had been admitted to an ICU. The median CCI score was 4 points (2–6). The median severity assessed by the APACHE II and SOFA scores was 17 points (17–22) and 5 points (2.25–8), respectively. Overall, 83 patients (46.1%) were infected with a single microbe, namely, Car-R/NonCar-S *P*. *aeruginosa*. Antimicrobial susceptibility testing of 180 Car-R/NonCar-S *P*. *aeruginosa* clinical isolates was performed, the results of which are shown in Table 1. Among the Car-R/NonCar-S *P*. *aeruginosa* clinical isolates (n = 180), only 7.22% of isolates were susceptible to imipenem and 19.4% to meropenem, 4.44% intermediate to imipenem and 12.22% to meropenem. Meanwhile 65% were susceptible to ceftazidime, 69.4% to cefepime, and 57.8% to ciprofloxacin. Of 179 clinical isolates, 61.5% were susceptible to piperacillin/tazobactam. The most common infections were in the lungs (43.9%), followed by the blood (26.1%). Overall, 180 cases of Car-R/NonCar-S *P*. *aeruginosa* infection, 89.4% received appropriate therapy. Among these group, 34.16% of participants received ceftazidime, which was the most common antibiotic used to treat Car-R/NonCar-S *P*. *aeruginosa*, followed by ciprofloxacin at a rate of 20.5%. The baseline characteristics of the participants and outcomes are listed in Table 2.

**Table 1:** Antimicrobial susceptibility of Car-R/NonCar-S *P*. *aeruginosa* (n = 180 isolates).

Antimicrobial Agents	Susceptibility (%)
Imipenem	13 (7.22)
Meropenem	35 (19.4)
Ceftazidime	117 (65)
Cefepime	125 (69.4)
Ciprofloxacin	104 (57.8)
Levofloxacin	80 (44.4)
Amikacin	151 (83.9)
Piperacillin/tazobactam (n = 179)^a^	110 (61.5)
Colistin (n = 179)^b^	178 (99.4)
Cefoperazone/sulbactam (n = 161)^a^	84 (52.2)
Gentamicin (n = 143)^a^	97 (67.8)
Ceftazidime/avibactam (n = 73)^a^	53 (72.6)
Ceftolozane/tazobactam (n = 65)^a^	42 (64.6)

^a^ Data on antimicrobial susceptibility were missing for a few isolates.

^b^ Isolates classified as “susceptible” or “intermediate” were considered susceptible to colistin.

**Table 2:** Characteristics and outcomes of patients with Car-R/NonCar-S *P*. *aeruginosa* infections (n = 180 cases).

Characteristic	Number (%)
Male, number (%)	118 (65.6)
Age (years), median (interquartile range; IQR)	73 (60–83)
Body weight (kg), median (IQR)	60.5 (52–70)
BMI (kg/m^2^), median (IQR)	23.19 (20.5–25.92)
Serum creatinine (mg/dL), median (IQR)	1.08 (0.71–1.88)
Creatinine clearance (ml/min), median (IQR)	49.16 (26.91–85.20)
Charlson comorbidity score (CCI), median (IQR)	4 (2–6)
Intensive-care unit	53 (29.4)
Septic shock, number (%)	24 (13.3)
Invasive devices, number (%)	150 (83.3)
APACHE II score, median, (IQR)	17 (12–22)
SOFA score, median (IQR)	5 (2.25–8)
PBS, median (IQR)	4 (2–6)
Sites of infection	
Pneumonia, number (%)	79 (43.9)
Bloodstream infections, number (%)	47 (26.1)
Skin and soft tissue infections, number (%)	17 (9.4)
Intraabdominal infections, number (%)	15 (8.3)
Urinary system infections, number (%)	7 (3.9)
Bone and joint infections, number (%)	5 (2.8)
Microbials	
Mono-microbial infections (*P*. *aeruginosa* only), number (%)	83 (46.1)
Poly-microbial infections, number (%)	97 (53.9)
Enterobacteriaceae	37 (38.1)
*Klebsiella pneumoniae*	36 (37.1)
*Acinetobacter baummanii*	19 (19.6)
*Stenotrophomonas maltophilia*	12 (12.4)
*Enterococcus* sp.	18 (18.6)
*Candida* sp.	4 (4.1)
Other gram-negative pathogens	7 (7.2)
Other gram-positive pathogens	5 (5.2)
Time to appropriate therapy (days), median (IQR)	2 (0–4)
Patients receive appropriate therapy within 48 hours, number (%)	161 (89.4)
Ceftazidime	55 (34.16)
Cefepime	8 (4.97)
Cefoperazone/sulbactam	3 (1.86)
Piperacillin/tazobactam	17 (10.56)
Ciprofloxacin	33 (20.50)
Levofloxacin	7 (4.35)
Gentamicin	1 (0.62)
Amikacin	9 (5.59)
Imipenem	3 (1.86)
Meropenem	5 (3.11)
Ceftazidime/avibactam	8 (4.97)
Ceftolozane/tazobactam	5 (3.11)
Colistin	8 (4.97)
Treatment outcomes	
14-day mortality	33 (18.3)
30-day mortality	52 (28.9)

APACHE II, Acute Physiology and Chronic Health Evaluation; SOFA, Sequential Organ Failure Assessment; PBS, Pitt bacteremia score

### Treatment outcomes

The 14-day and 30-day mortality rates were 18.3% and 28.9%, respectively. Treatment outcomes are shown in Table 2.

### Antibiotic regimen and treatment outcome

**Fig 1:**
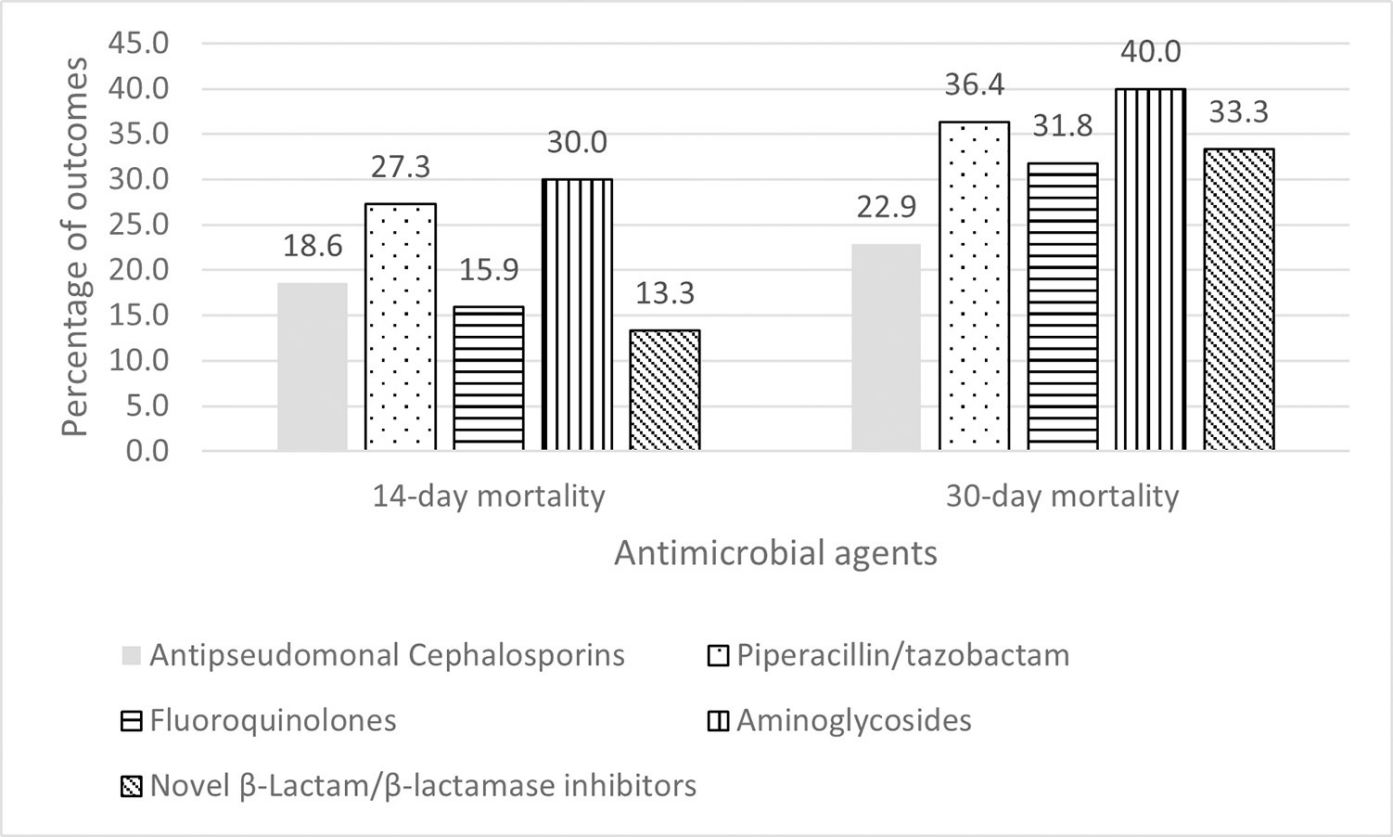
Rates of 14-day and 30-day mortality with each regimen. Antipseudomonal cephalosporins: ceftazidime, cefepime, cefoperazone/sulbactam; fluoroquinolones: ciprofloxacin, levofloxacin; aminoglycosides: gentamicin, amikacin; novel β- lactam/β-lactamase inhibitors: ceftazidime/avibactam, ceftolozane/tazobactam.

Patients who received aminoglycoside and piperacillin/tazobactam appeared to have higher 14-day and 30-day mortality rates than those with other antipseudomonal antimicrobial agents, as shown in Fig 1. The results also showed that traditional β-lactams would increase 14-day mortality compared with fluoroquinolones, which can probably be explained by patients receiving piperacillin/tazobactam having a higher 14-day mortality rate than those receiving ceftazidime or fluoroquinolones, as shown in S1 Table.

Regarding 30-day mortality, this showed the same result, with a trend of this rate being higher in patients receiving piperacillin/tazobactam than in those with ceftazidime or fluoroquinolones.

### Factors associated with 14-day and 30-day mortality

Based on the mortality results for the different antibiotic regimens, we analyzed the administration of piperacillin/tazobactam as one of the risk factors associated with 14-day and 30-day mortality. From logistic regression analysis, the factors significantly associated with 14-day mortality were bloodstream infection (OR 1.97, 95% CI 0.88–4.43), septic shock (OR 3.3, 95% CI 1.30–8.40), APACHE II < 14 (OR 0.13, 95% CI 0.03–0.54), SOFA < 7 (OR 0.25, 95% CI 0.11–0.56), and PBS < 4 (OR 0.16, 95% CI 0.05–0.47). In multivariable analysis, PBS < 4 was associated with 14-day mortality (OR 0.23, 95% CI 0.07–0.76), as shown in Table 3.

**Table 3:** Factors associated with 14-day mortality.

Variables	14-day mortality			
	Univariate analysis		Multivariate analysis	
	OR (95%CI)	*P* value	OR (95%CI)	*P* value
Age <65 years (n = 64)	0.53 (0.22–1.25)	0.22		
Admission in intensive-care unit (n = 53)	1.48 (0.67–3.27)	0.34		
Bloodstream infections (n = 47)	1.97 (0.88–4.43)	0.095	2.24 (0.94–5.33)	0.07
Presence of invasive devices (n = 150)	2.25 (0.64–7.92)	0.20		
Septic shock (n = 24)	3.30 (1.30–8.40)	0.009	1.36 (0.46–4.06)	0.58
CCI (<4) (n = 76)	0.63 (0.29–1.40)	0.26		
APACHE II (<14) (n = 52)	0.13 (0.03–0.54)	0.001	0.28 (0.06–1.35)	0.11
SOFA (<7) (n = 114)	0.25 (0.11–0.56)	<0.001	0.57 (0.23–1.38)	0.21
PBS (<4) (n = 73)	0.16 (0.05–0.47)	<0.001	0.23 (0.07–0.76)	0.02
Receiving appropriate antibiotic within 48 hours (n = 161)	0.82 (0.26–2.67)	0.75		
Piperacillin/tazobactam used (n = 22)	1.82 (0.65–5.07)	0.25		

APACHE II, Acute Physiology and Chronic Health Evaluation; SOFA, Sequential Organ Failure Assessment; PBS, Pitt bacteremia score; OR, odds ratio; CI, confidence interval

Logistic regression analysis also showed that admission to an ICU (OR 0.43, 95% CI 0.18–1.04), septic shock (OR 2.74, 95% CI 0.91–8.13), PBS <4 (OR 0.35, 95% CI 0.13–0.97), and receiving appropriate treatment within 48 h (OR 0.35, 95% CI 0.11–1.12) were significantly associated with 30-day mortality. However, in multivariable analysis, only septic shock and PBS <4 were associated with 30-day mortality, as shown in S2 Table. The data showed a trend for piperacillin/tazobactam used to be associated with increases in both 14-day and 30-day mortality, although these were not significant.

## Discussion

Few studies globally have reported on the characteristics, treatment outcomes, and factors associated with 14-day mortality of Car-R/NonCar-S *P*. *aeruginosa* infection. Most studies of this kind that have been performed were retrospective in nature and included a small group of patients. According to the limited evidence regarding its treatment, it is challenging to determine the optimal treatment option for Car-R/NonCar-S *P*. *aeruginosa* infection, even though clinicians are likely to encounter such pathogens on a regular basis.

The rate of 14-day mortality in our study was 18.3%, similar to the finding in a previous study by Buehrle et al. (19%) [4]. In the same study, the Pitt bacteremia score was the only factor significantly associated with 14-day mortality in multivariate analysis, which matches the result in our study. Upon comparison with previous studies [4, 6–12], ours is the first to demonstrate risk factors of 14-day mortality in Car-R/NonCar-S *P*. *aeruginosa* infection and confirmed that higher severity of infection had increased risk of mortality.

Balkhair et al. [5] reported the 30-day mortality rate in *P*. *aeruginosa*-infected patients and found that it differed between deaths upon healthcare-associated infection with carbapenem-sensitive *P*. *aeruginosa* (22.6%) and carbapenem-resistant *P*. *aeruginosa* (60.1%). In our study, the 30-day mortality rate was 28.3%, which is similar to the rate in patients with carbapenem-sensitive *P*. *aeruginosa*. Infections by multidrug-resistant microorganisms would be expected to be associated with higher mortality than those with susceptible strains [22]. Since Car-R/NonCar-S *P*. *aeruginosa* is still susceptible to antipseudomonal β-lactam agents other than carbapenem, patients most likely received an effective antimicrobial at an early stage of infection, reducing the mortality relative to that in patients with carbapenem-resistant *P*. *aeruginosa* infection. This highlights the importance of the time until an appropriate antimicrobial is administered.

In multivariable analysis, we found that admission to an ICU, septic shock, PBS < 4, and receiving appropriate treatment within 48 h were associated with 30-day mortality, which is similar to the findings in previous studies [10, 12]. Montero et al. [12] found that pneumonia, septic shock, PBS, and inappropriate definitive therapy, were associated with 30-day mortality in patients infected with extensively drug-resistant *P*. *aeruginosa*. Moreover, we found that all cases of a failure to receive appropriate antibiotics in our study were due to therapy delay, leading to more deaths in hospital. This is consistent with a previous study by Dantas et al. [10], who studied 120 patients with *P*. *aeruginosa* bacteremia and reported that inappropriate initial antimicrobial therapy was associated with 30-day mortality. We also reported that the group of patients in whom effective antimicrobial therapy was delayed had a higher rate of 30-day mortality. These results consistently showed a trend toward higher mortality as the length of this delay increased, emphasizing the importance of the timely initiation of an effective antibiotic and the severity of infection in the outcome of Car-R/NonCar-S *P*. *aeruginosa* infection.

For CRPA that is still susceptible to antipseudomonal β-lactam agents, the IDSA [18] suggests that antipseudomonal β-lactam agents be used, but to the best of our knowledge no study has confirmed the superiority of any particular therapy in this context. Only a few studies have assessed the efficacy of each choice of therapy. For example, Khalili et al. [1] found no difference in the cure rate in 10 patients who received cephalosporin to treat carbapenem-resistant/cephalosporin-susceptible *P*. *aeruginosa* infection. In addition, Ng et al. [19] evaluated 87 patients diagnosed with carbapenem-resistant/cephalosporin-susceptible *P*. *aeruginosa* pneumonia and found no difference in 30-day in hospital mortality between those treated with cefepime and those with other antipseudomonal agents to which *P*. *aeruginosa* is susceptible. Our study included more participants, and comparison among the groups of antipseudomonal agents could be performed. The results resembled those obtained elsewhere in showing that there was no significant difference in 14-day and 30-day mortality among the regimens, although we found a tendency for treatment with piperacillin/tazobactam to have a negative effect on mortality.

Overall, 36% of patients who received piperacillin/tazobactam died within 30 days. Piperacillin/tazobactam use is one of the factors that showed a trend toward increasing 14-day and 30-day mortality. Yoshida et al. [23] also found that critically ill patients with CRPA who had been treated with piperacillin/tazobactam exhibited clinical failure more often. The authors were concerned that this might have been due to the piperacillin concentration varying in critically ill patients, leading to subtherapeutic treatment. Most of the patients in our study had APACHE score ≥ 14 (71%), which represents a high severity of infection. Additionally, we found that 5 out of 8 patients who received piperacillin/tazobactam and died within 30 days had a level of susceptibility to piperacillin/tazobactam reported as “intermediate.” The probability of subtherapeutic treatment upon the use of piperacillin/tazobactam in severe patients with high MIC of Car-R/NonCar-S *P*. *aeruginosa* would explain the outcome of our study. A study by Gill et al. [24] also evaluated the dose of piperacillin/tazobactam against CRPA by using Monte Carlo simulation. It revealed that CRPA had an elevated MIC for piperacillin/tazobactam, 71% had an MIC of 8/4 to 16/4 μg/mL, and needed 4.5 g of piperacillin/tazobactam for 4 h every 6 h to optimize attainment of the pharmacokinetic/pharmacodynamic (PK/PD) target. For an isolate with an MIC of 32/4 μg/mL, which would be categorized as “intermediate” according to CLSI guideline version 2023 [25], 4.5 g of piperacillin/tazobactam for 4 h every 6 h can attained a 60% probability of target attainment (PTA) and 10% PTA at MIC 32/4 μg/mL and 64/4 μg/mL, respectively of 50% time over MIC [26]. Since 2023, CLSI has updated the MIC breakpoint for *P*. *aeruginosa* in the intermediate group from 32/4–64/4 to 32/4 μg/mL. Meanwhile, our study included patients from as early as 2019, which means that some of the patients who received piperacillin/tazobactam might have had an MIC as high as 64/4 μg/mL and their target attainment probably could not have been optimized despite receiving 4.5 g of piperacillin/tazobactam for 4 h every 6 h. This might explain the higher 14-day and 30-day mortality rates.

A limitation of our study is that we did not have the true MIC of piperacillin/tazobactam in each patient to prove the above hypothesis. Nonetheless, our results emphasize the importance of MIC, which can lead to an appropriate dosing regimen for patients infected with Car-R/NonCar-S *P*. *aeruginosa*. Moreover, clinicians should be aware of the possibility of deep-seated infection or infection without source control. Harada et al. [27] studied the *in vitro* inoculum effect on piperacillin/tazobactam and found that the MIC_50_ of piperacillin/tazobactam would increase eightfold in the high inoculum (10^7^ CFU/mL), making it more difficult for piperacillin/tazobactam to attain the PK/PD target at the site of infection. If the Car-R/NonCar-S *P*. *aeruginosa* displays “intermediate” susceptibility to piperacillin/tazobactam, we do not suggest piperacillin/tazobactam due to its lower efficacy in a high-inoculum model, especially in patients with deep-seated infection or infection without source control.

We also found that, even though Car-R/NonCar-S *P*. *aeruginosa* was still susceptible to piperacillin/tazobactam, it was resistant to almost all other antipseudomonal β-lactam drugs, including ceftazidime/avibactam and ceftolozane/tazobactam. Although we did not test the resistance mechanism in our study, these susceptibility patterns revealed that Car-R/NonCar-S *P*. *aeruginosa* might carry metallo-β-lactamase, which is capable of hydrolyzing all classes of β-lactam drugs. A study by Vallabhaneni et al. [28] that used antimicrobial susceptibility testing to identify carbapenemase-producing *P*. *aeruginosa* found that, among CRPA isolates, adding the criterion of a lack of susceptibility to cefepime or ceftazidime can be used to identify carbapenemase-producing CRPA isolate with sensitivity of 91% and specificity of 50%. Moreover, adding the criterion of a lack of susceptibility to ceftolozane/tazobactam could be even more valuable in identifying carbapenemase-producing CRPA, with 100% sensitivity and 86% specificity. The result of our study that patients treated with piperacillin/tazobactam had worse outcomes could potentially be explained by the causative pathogen carrying metallo-β-lactamase. The fact that the pathogen was still susceptible to piperacillin/tazobactam regarding current MIC breakpoint of *P*. *aeruginosa* quite high. The MIC breakpoint of piperacillin/tazobactam may be revised as same as in Enterobacterales [29].

Patients who received aminoglycosides had higher rates of 14-day and 30-day mortality, but these were not significantly different from those of patients who received traditional β-lactams. Since 2023, the CLSI had made a major change to the aminoglycoside breakpoint for *P*. *aeruginosa*. Specifically, in CLSI M100 edition 2023, the breakpoint of gentamicin has been removed from *P*. *aeruginosa* and amikacin is suggested only for urinary tract infections. This is due to the rise of resistance to gentamicin and the fact that amikacin can only achieve bacteriostasis, making it suitable only for conditions with a lower bacterial burden with good source control, such as urinary tract infections [30]. In our study, we collected data since 2019, at which time aminoglycosides were still one of the choice therapies. Indeed, in our study, 5 of 8 patients received amikacin to treat bacteremia, which might not have been suitable and would have explained the higher rate of mortality in these patients. We support the update of CLSI 2023 that aminoglycosides should be used only in urinary tract infections.

The results of this study support the use of traditional β-lactams to treat Car-R/NonCar-S *P*. *aeruginosa* after susceptibility to this group of drugs has been confirmed, in accordance with the IDSA guidance [18]. Specifically, this support is due to there being no difference between each regimen in the 14-day and 30-day mortality rates. For piperacillin/tazobactam, since it showed a trend of negatively affecting mortality, clinicians would use piperacillin/tazobactam only in certain selected cases with MIC less than 16/4 μg/mL in low inoculum site of infection [27] and source control.

This study has some limitations. First, the number of patients who received piperacillin/tazobactam was small, so further study focusing on piperacillin/tazobactam used in CRPA infection might be needed to establish the true efficacy of these agents. Second, we did not study the mechanism by which pathogens achieve resistance, which affect the decision of antipseudomonal regimen. Finally, the majority of our participants had polymicrobial infection (53.9%), which would have influenced the mortality rates and obscured the true effects of antimicrobials.

## Conclusion

Antipseudomonal β-lactam agents can be used to treat Car-R/NonCar-S *P*. *aeruginosa*. To make the optimal choice of treatment and improve clinical outcomes, we should consider age, co-morbidities, disease severity, and appropriate antimicrobials. Although there is concern about different outcomes between antipseudomonal β-lactam agents, antipseudomonal cephalosporins and fluoroquinolone can be used to treat Car-R/NonCar-S *P*. *aeruginosa*, but the use of piperacillin/tazobactam in Car-R/NonCar-S *P*. *aeruginosa* infections might need further investigation.

## Supporting information

S1 Table Antibiotic regimens and associated 14-day and 30-day mortality. OR, odds ratio; CI, confidence interval.
(DOCX)

S2 Table Factors associated with 30-day mortality. APACHE II, Acute Physiology and Chronic Health Evaluation; SOFA, Sequential Organ Failure Assessment; PBS, Pitt bacteremia score; OR, odds ratio; CI, confidence interval.
(DOCX)
