# Supplementary material for: Evaluation of risk factors for 14-day and 30-day mortality among treatment regimens against Pseudomonas aeruginosa resistant to carbapenem but susceptible to traditional antipseudomonal non-carbapenem β-lactam agents
Source: PLoS One. 2024 Nov 19;19(11):e0313944. doi: 10.1371/journal.pone.0313944 (PMC11575795; doi:10.1371/journal.pone.0313944)
Supplement: S1 Table — OR, odds ratio; CI, confidence interval. (DOCX) [file pone.0313944.s001.docx]

# **Supporting information**

**S1 Table. Antibiotic regimens and associated 14-day and 30-day mortality.**

| Variables | 14-day mortality | | 30-day mortality | |
| --- | --- | --- | --- | --- |
|  | **OR (95%CI)** | ***P* value** | **OR (95%CI)** | **OR (95%CI)** |
| Traditional β-lactams VS fluoroquinolones (n=94 VS 43) | 1.30 (0.50-3.38) | 0.65 | 0.79 (0.36-1.73) | 0.69 |
| Traditional β-lactams VS active carbapenems (n=95 VS 8) | 1.75 (0.20-15.10) | 1.00 | 2.64 (0.31-22.49) | 0.68 |
| Traditional β-lactams VS aminoglycosides (n=95 VS 10) | 0.58 (0.14-2.47) | 0.43 | 0.57 (0.15-2.17) | 0.47 |
| Traditional β-lactams VS novel cephalosporins (n=95 VS 15) | 1.63 (0.34-7.82) | 0.73 | 0.75 (0.24-2.42) | 0.76 |
| Traditional cephalosporin VS piperacillin/tazobactam (n=73 VS 22) | 0.58 (0.19-1.76) | 0.37 | 0.57 (0.21-1.59) | 0.41 |
| Ceftazidime VS piperacillin/tazobactam (n=62 VS 22) | 0.51 (0.16-1.63) | 0.34 | 0.42 (0.14-1.23) | 0.15 |
| Piperacillin/tazobactam VS fluoroquinolones (n=22 VS 44) | 1.98 (0.58-6.84) | 0.33 | 1.22 (0.42-3.59) | 0.79 |

OR, odd ratio; CI, confidence interval
