# Supplementary material for: Evaluation of risk factors for 14-day and 30-day mortality among treatment regimens against Pseudomonas aeruginosa resistant to carbapenem but susceptible to traditional antipseudomonal non-carbapenem β-lactam agents
Source: PLoS One. 2024 Nov 19;19(11):e0313944. doi: 10.1371/journal.pone.0313944 (PMC11575795; doi:10.1371/journal.pone.0313944)
Supplement: S2 Table — APACHE II, Acute Physiology and Chronic Health Evaluation; SOFA, Sequential Organ Failure Assessment; PBS, Pitt bacteremia score; OR, odds ratio; CI, confidence interval. (DOCX) [file pone.0313944.s002.docx]

# **Supporting information**

S2 Table. **Factors associated with 30-day mortality.**

| Variables | 30-day mortality | | | |
| --- | --- | --- | --- | --- |
|  | **Univariate analysis** | | **Multivariate analysis** | |
|  | **OR (95%CI)** | ***P* value** | **OR (95%CI)** | ***P* value** |
| Age <65 years (n=64) | 0.61 (0.16-2.35) | 0.47 |  |  |
| Admission in intensive-care unit (n=53) | 0.43 (0.18-1.04) | 0.06 | 0.50 (0.22-1.15) | 0.10 |
| Bloodstream infections (n=47) | 1.63 (0.67-4.01) | 0.29 |  |  |
| Presence of invasive devices (n=150) | 0.94 (0.31-2.81) | 0.91 |  |  |
| Septic shock (n=24) | 2.74 (0.91-8.13) | 0.07 | 3.23 (1.24-8.42) | 0.02 |
| CCI (<4) (n=76) | 0.72 (0.37-1.39) | 0.33 |  |  |
| APACHE II (<14) (n=52) | 0.81 (0.27-2.44) | 0.71 |  |  |
| SOFA (<7) (n=114) | 0.64 (0.26-1.59) | 0.34 |  |  |
| PBS (<4) (n=73) | 0.35 (0.13-0.97) | 0.04 | 0.31 (0.14-0.69) | 0.004 |
| Receiving appropriate antibiotic within 48 hours (n=161) | 0.35 (0.11-1.12) | 0.08 | 0.42 (0.14-1.23) | 0.11 |
| Piperacillin/tazobactam used(n=22) | 1.20 (0.42-3.43) | 0.73 |  |  |

APACHE II, Acute Physiology and Chronic Health Evaluation; SOFA, Sequential Organ Failure Assessment; PBS, Pitt bacteremia score; OR, odds ratio; CI, confidence interval
